# Alveolar bone heights of maxillary central incisors in unilateral cleft lip and palate patients using cone-beam computed tomography evaluation

**DOI:** 10.1007/s00056-020-00276-w

**Published:** 2021-02-05

**Authors:** Marcin Stasiak, Anna Wojtaszek-Słomińska, Bogna Racka-Pilszak

**Affiliations:** Department of Orthodontics, Faculty of Medicine, Medical University of Gdańsk, Aleja Zwycięstwa 42c, 80-210 Gdańsk, Poland

**Keywords:** Alveolar bone grafting, Cleft lip, Cleft palate, Orthodontic treatment, Retrospective studies, Alveolarknochentransplantation, Lippenspalte, Gaumenspalte, Kieferorthopädische Behandlung, Retrospektive Studien

## Abstract

**Purpose:**

The aims of this retrospective cross-sectional study were to measure and compare labial and palatal alveolar bone heights of maxillary central incisors in unilateral cleft lip and palate patients, following STROBE (Strengthening the Reporting of Observational Studies in Epidemiology) guidelines.

**Patients and methods:**

The study group consisted of 21 patients with a mean age of 16 years. High-resolution cone-beam computed tomography was performed at least one year after secondary alveolar bone grafting. The experimental side was the cleft side and the contralateral side without congenital cleft was the control. Measurements were performed on incisors’ midsagittal cross-sections. The Wilcoxon signed-rank test was used for intergroup comparisons.

**Results:**

The labial and palatal distances between alveolar bone crests and cementoenamel junctions were significantly greater on the cleft side than on the noncleft side. Mean differences were 0.75 and 1.41 mm, respectively. The prevalence of dehiscences at the cleft side maxillary central incisors was 52% on the labial surface and 43% on the palatal surface. In the controls, it was 19% and 14%, respectively.

**Conclusion:**

The cleft-adjacent maxillary central incisors had more apically displaced alveolar bone crests on the labial and palatal sides of the roots than the controls. Higher prevalence of dehiscences was found on the cleft side. Bone margin differences predispose to gingival height differences of the central incisors. These differences could increase the demands of patients to obtain more esthetic treatment results with orthodontic extrusion and periodontal intervention on the cleft side.

**Supplementary Information:**

The online version of this article (10.1007/s00056-020-00276-w) contains supplementary information, which is available to authorized users.

## Abstract (de)

**Zielsetzung:**

Ziele dieser retrospektiven Querschnittsstudie waren die Messung und der Vergleich der labialen und palatinalen Alveolarknochenhöhen der oberen zentralen Schneidezähne bei Patienten mit unilateralen Lippen-Kiefer-Gaumen-Spalten gemäß den STROBE(„Strengthening the Reporting of Observational Studies in Epidemiology“)-Richtlinien.

**Patienten und Methoden:**

Die Studienteilnehmergruppe bestand aus 21 Patienten mit einem Durchschnittsalter von 16 Jahren. Die hochauflösende digitale Volumentomographie wurde frühestens ein Jahr nach der sekundären Alveolarknochentransplantation durchgeführt. Die experimentelle Seite war die Spaltseite, die kontralaterale Seite ohne kongenitale Spaltbildung diente als Kontrolle. Die Messungen wurden an den mittsagittalen Querschnitten der Schneidezähne durchgeführt. Für Vergleiche zwischen den Gruppen wurde der Vorzeichen-Rang-Test nach Wilcoxon verwendet.

**Ergebnisse:**

Die labialen und palatinalen Abstände zwischen den Alveolarkämmen und der Schmelz-Zement-Grenze waren auf der Spaltseite signifikant größer als auf der Nicht-Spaltseite. Die mittleren Unterschiede betrugen 0,75 bzw. 1,41 mm. Die Prävalenz von Dehiszenzen an den spaltseitigen oberen zentralen Schneidezähnen betrug 52% auf der labialen und 43% auf der palatinalen Fläche. Bei den Kontrollen lag sie bei 19 bzw. 14%.

**Schlussfolgerung:**

Die spaltseitigen oberen zentralen Schneidezähne hatten mehr apikal verlagerte Alveolarkämme auf der labialen und palatinalen Seite der Wurzeln als die Kontrollen. Eine höhere Prävalenz von Dehiszenzen wurde auf der Spaltseite gefunden. Knochenranddifferenzen prädisponieren zu gingivalen Höhenunterschieden der zentralen Schneidezähne. Diese Unterschiede könnten die Ansprüche von Patienten an die Behandlung erhöhen, durch eine kieferorthopädische Extrusion und paradontale Intervention auf der Spaltseite ein noch ansprechenderes ästhetisches Behandlungsergebnis zu erzielen.

## Introduction

Cleft lip and palate (CLP) represents one of the most common congenital conditions in the facial segment of the cranium [30]. A characteristic feature of clefts includes partial or complete lack of anatomical tissue continuity and tissue hypoplasia in the affected area. Cleft is a developmental malformation that results from both genetic and environmental factors [29].

Treatment of CLP patients is an interdisciplinary and long-term process [19]. The rehabilitation protocol includes secondary alveolar bone grafting (SABG) performed when the patient reaches mixed dentition [4]. The purpose of the autogenous bone grafting is closure of the oro-nasal fistula and obtainment of anatomical tissue continuity of the alveolar process in the maxilla [1].

Bone transplant results must be evaluated before continuing orthodontic treatment following alveolar grafting. When a lateral incisor is missing (frequent condition in CLP patients [16]), these findings help specialists decide whether the tooth should be restored or the space should be closed [21]. Interproximal alveolar bone height measurements on the root surfaces of the cleft adjacent teeth were widely used for SABG assessment on the basis of two-dimensional (2D) x‑ray images and 2D cross-sections from three-dimensional (3D) images [21].

Moreover, it seems that bone bridge assessment and identification of the bony support of the teeth associated with cleft is crucial for the further therapy. CLP patients are at high risk for periodontal disease progression, and cleft sites suffered more periodontal tissue destruction than the noncleft sites in these patients [20]. Esthetic issues like gingival recessions that appear secondary to the alveolar bone defects [6] or to camouflage treatment of the skeletal discrepancy in CLP patients by labial movement and proclination of incisors [12] justify careful assessment of labial and palatal alveolar bone heights. These measurements are not possible with conventional 2D x‑rays.

Three-dimensional x‑ray diagnostics is an appropriate tool for alveolar bone analysis [21, 22]. As radiological protection is needed for this type of examination (especially in young patients), it seems justified to use cone-beam computed tomography (CBCT) instead of computed tomography (CT) examination [21, 31]. Furthermore, a small field of view is recommended if possible [21]. There is no need to perform additional 3D x‑ray images because those routinely performed for alveolar bone grafting assessment can be utilized for alveolar bone height measurements.

Up to now, only one paper evaluated both labial and palatal alveolar bone heights of maxillary central incisors in CLP patients using CBCT [27]. Thus, we decided to perform this retrospective observational cross-sectional study to measure and to compare labial and palatal alveolar bone heights of maxillary central incisors in complete unilateral cleft lip and palate (UCLP) patients treated in the same orthodontic department. The null hypothesis was that the maxillary central incisors have the same labial and palatal alveolar bone heights on the cleft and noncleft sides in UCLP patients.

## Patients and methods

Strengthening the Reporting of Observational Studies in Epidemiology (STROBE) guidelines were used in this study [7]. CLP patients are treated according to a complex protocol. For ethical reasons, it is not possible to obtain an untreated control group. Therefore, a split-mouth study design was selected. The experimental side was the cleft side, and the control side was the contralateral side with normal anatomy. The study design was approved by the Ethics Committee of the Medical University of Gdańsk (approval number NKBBN/311/2017).

The research was conducted in the orthodontic department. The department has been utilizing 3D x‑ray imaging since 2017. CBCT with a small field of view is an element of standard medical documentation in CLP patients after alveolar bone grafting. Patient sampling was performed from July 2018 to October 2018. Two patients underwent surgery in February 2005 and October 2006. The remainder underwent surgery from August 2011 to June 2017. Radiographs were taken from July 2017 to September 2018 [22]. Measurements were performed from March to June 2019.

Eligibility criteria were as follows: Complete UCLP without other congenital deformities, SABG surgery, and CBCT at least one year after grafting. Bilateral clefts were not included in the study due to the inability to compare contralateral sites. Unilateral cleft lip and alveolus (UCLA) patients were also excluded due to qualitative reasons.

In the first stage of selection, all patients currently treated in the orthodontic department with complete UCLP recognition were identified by means of an electronic medical records software (Estomed; Hakon Software, Gdańsk, Poland). Subsequently, patients were examined and qualified during their orthodontic appointments by all authors. Analysis of the medical documentation was performed, and all patients who met the eligibility criteria were included in the study.

Study outcomes were quantitative measurements of the labial alveolar bone height (LABH) and the palatal alveolar bone height (PABH) distances based on CBCT examinations. Patients differed in their follow-up periods and orthodontic treatment stages. Potential confounders were artefacts due to the metal fixed orthodontic appliances [25].

**Fig. 1:**
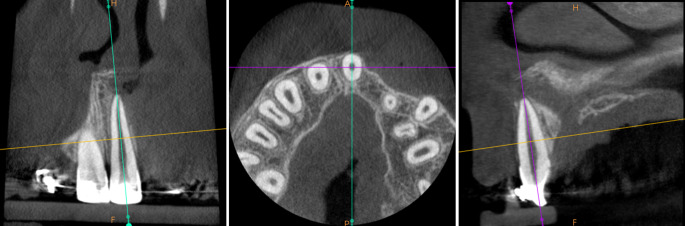
Standardized cone beam computed tomographic image Standardisierte DVT(digitale Volumentomographie)-Aufnahme

**Fig. 2:**
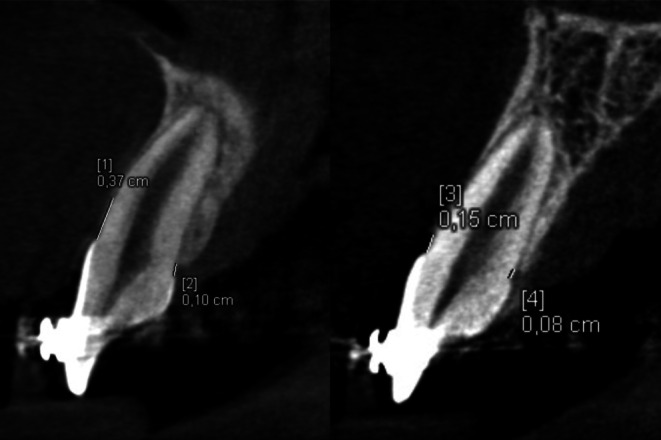
Measurement of the labial and palatal alveolar bone heights on midsagittal cross-sections of the cleft (*left*) and the noncleft (*right*) maxillary central incisors Messung der labialen und palatinalen Alveolarknochenhöhen auf mittsagittalen Querschnitten der oberen zentralen Schneidezähne, *links* mit Spalte, *rechts* ohne Spalte

The high-resolution CBCT examinations were performed with a CS 8100 3D scanner (Carestream, Atlanta, GA, USA). The imaging conditions were: 80 kV, 5 mA, 12 s, voxel size of 0.2 mm, field of view (FOV) of 5 cm × 5 cm. The images were analyzed by means of the Syngo.via software (Siemens Healthcare, Erlangen, Germany). Standardization was obtained after reorientation of the images according to the long axes of the central incisors (Fig. 1). The labial and palatal alveolar bone heights were measured on incisors’ midsagittal cross-sections, both on the cleft and noncleft sides, by the first author, who is experienced in CLP patients’ treatment. The cementoenamel junctions were points of reference. To precisely set the bone bridge crest and the cementoenamel junction’s positions, the evaluation was performed at the same time on the midsagittal and horizontal cross-sections. The bone bridge crest point was set at the lowest point of the bone on the central incisor’s surface. The cementoenamel junction point was set in the most apical point of the enamel (Fig. 2). Measurements were performed twice with an interval of at least 2 months. Arithmetic means were used for further comparative analyses. A critical amount for dehiscence on the CBCT was defined to be 2 mm [23].

Bias resulting from incisors tipping and angulation was eliminated by standardized reorientation of the images. Measurements were performed twice with an interval of two months, and arithmetic means were used for further analyses. Therefore, potential bias from imprecision was reduced. Follow-up differences do not seem to play a significant role because autogenous bone grafts show some stabilization after one year [9]. Moreover, on account of age factors, the influence of age-related periodontal atrophy could be discounted.

Database was collected in Microsoft Excel file (Microsoft, Redmond, WA, USA). Statistical analyses were performed with TIBCO Statistica™ software (version 13.3, StatSoft, Tulsa, OK, USA) and RStudio software (version 3.6.0, RStudio, Boston, MA, USA). Normal distribution was assessed by means of Shapiro–Wilk test. Spearman’s rank correlation coefficient was used for intrarater reproducibility measurements. Wilcoxon signed-rank test was used for experimental side and control side comparisons. The latter two statistical tests were chosen due to the former test’s results. A bootstrapping analysis in RStudio was used for test power calculation.

## Results

In the first stage of selection, 62 patients were identified by means of the electronic medical records software. Patients were excluded due to no bone grafting—before (*n* = 26) or not qualified (*n* = 5), primary bone grafting (*n* = 6), tertiary bone grafting (*n* = 2) and lack of CBCT examination with required follow-up period (*n* = 2). A total of 21 patients were confirmed eligible and further analyzed.

The study group consisted of 5 (24%) female and 16 (76%) male patients. In 9 (43%) cases the cleft was identified on the right and in 12 (57%) cases on the left side. The lateral incisor was missing in 11 (52%) patients. An equal number of the patients presented with the lateral incisors in the major and in the minor segments (*n* = 5). The bone bridge was obtained in 15 (71%) patients after SABG [22]. Study group characteristics are presented in Table 1 [22].

**Table 1:** Study group characteristics (from: [22], Open Access published under Creative Commons, Version 4.0, CC BY 4.0; the material was not modified) Charakteristik des Studienkollektivs

	Mean (SD)	Median	Min–max
SABG age, years	10.96 (1.81)	11.06	6.91–14.09
CBCT age, years	16.15 (2.84)	15.68	11.66–21.18
Follow-up, years	5.19 (2.75)	5.34	1.18–12.43

*SD* standard deviation, *Min–max* Minimum–maximum, *SABG* secondary alveolar bone grafting, *CBCT* cone-beam computed tomography

Patients underwent operations in five rehabilitation centers by five different plastic surgeons according to the Boyne and Sands technique [4]. Preoperatively, the upper arch was expanded, and teeth were aligned using fixed orthodontic appliances. Palatal expansion did not lead to diastema occurrence in the UCLP patients before grafting. It resulted in translocation of the minor and major bone segments, palatal soft tissue straining, and cleft space extension. Iliac crest bone grafts were used in 17 patients before the eruption of the canine and in 4 patients before the eruption of the lateral incisor. Orthodontic treatment was continued after the surgery.

**Fig. 3:**
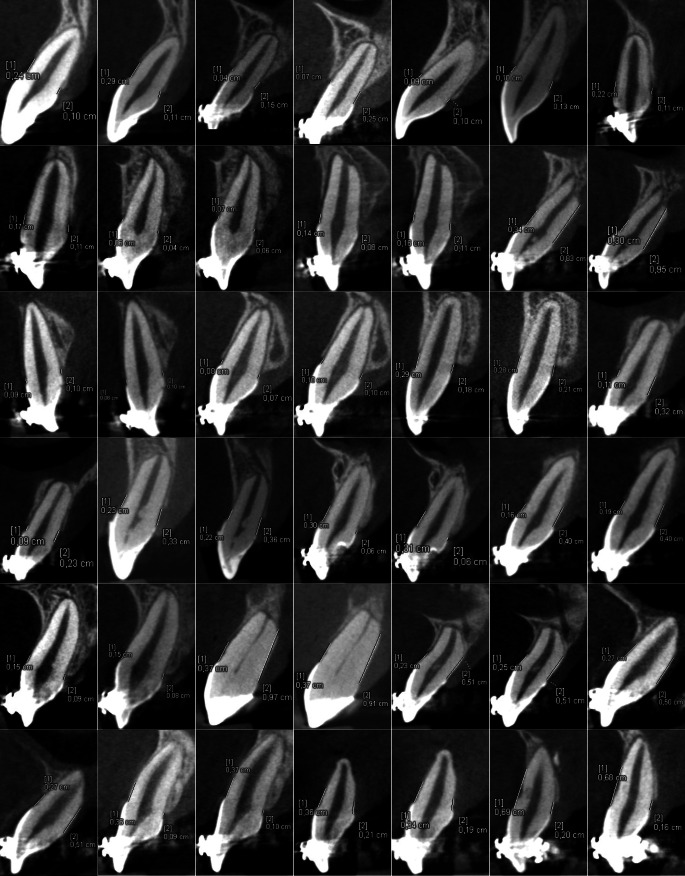
Cross-sectional images of both cleft side measurement series in all patients Querschnittsbilder beider Messreihen der Spaltseite bei allen Patienten

**Fig. 4:**
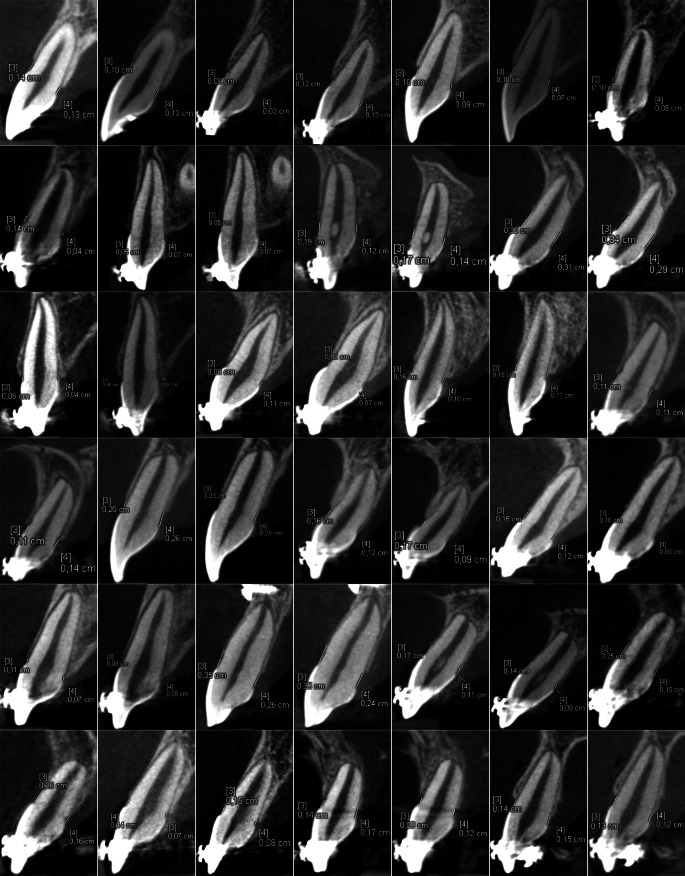
Cross-sectional images of both noncleft side measurement series in all patients Querschnittsbilder beider Messreihen der Seite ohne Spalte bei allen Patienten

**Fig. 5:**
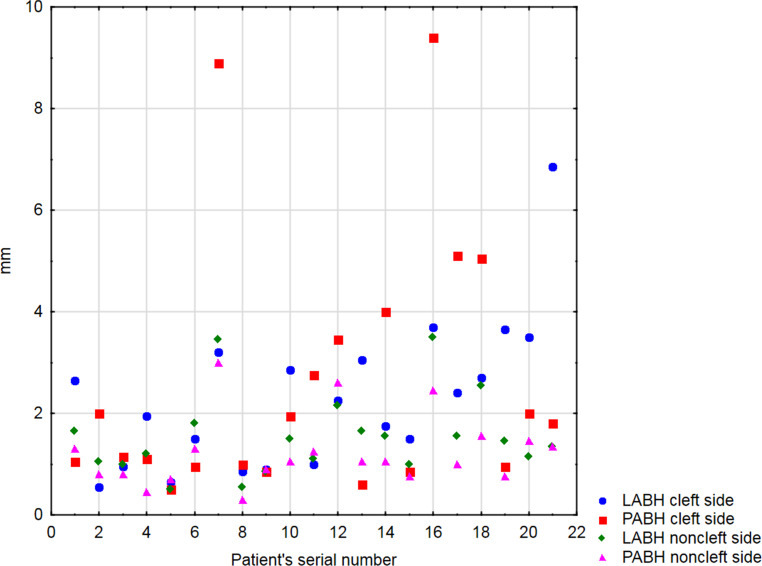
Scatter plot with arithmetic means of the collected data in all patients. *LABH* labial alveolar bone height, *PABH* palatal alveolar bone height, *mm* millimeters Scatter-Plot mit arithmetischen Mittelwerten der erhobenen Daten aller Patienten. *LABH* labiale Alveolarknochenhöhe, *PABH* palatinale Alveolarknochenhöhe, *mm *Millimeter

No metal artefacts preventing bone evaluation were noticed. LABH and PABH measurements on the cleft and noncleft sides were performed twice in all patients (Fig. 3 and 4). The arithmetic means of the collected data (Supplementary Table) are shown in the scatter plot (Fig. 5). LABH measurements obtained results from 0.55 to 6.85 mm and PABH measurements from 0.5 to 9.4 mm on the cleft side. On the noncleft side, these values were 0.5–3.5 mm and 0.3–3.0 mm, respectively. In our study, 11 (52%) patients demonstrated dehiscences on the labial surface and 9 (43%) on the palatal surface of the cleft side central incisors. In the controls, this finding appeared in 4 (19%) and 3 (14%) patients, respectively.

In all statistical measurements, a 95% confidence interval was adopted. Shapiro–Wilk test revealed that the evaluated variables were not normally distributed (*p* < 0.05; Table 2). For that reason, nonparametric tests were used for subsequent analyses.

**Table 2:** Normal distribution assessment by means of Shapiro–Wilk test Assessment der Normalverteilung anhand des Shapiro-Wilk-Tests

Measurement	*p*
LABH cleft side	0.014973*
PABH cleft side	0.000146*
LABH noncleft side	0.008748*
PABH noncleft side	0.006030*

*LABH* labial alveolar bone height, *PABH* palatal alveolar bone height

*Statistically significant at *p* < 0.05

The Spearman’s rank correlation coefficient obtained results from 0.75 to 0.98 and thus revealed high and statistically significant (*p* < 0.05) intrarater reproducibility (Table 3).

**Table 3:** Intrarater reproducibility according to Spearman’s rank correlation coefficient Intrarater-Reproduzierbarkeit nach dem Spearman-Rangkorrelationskoeffizienten

Measurement	R	*p*
LABH cleft side	0.981783	0.000000*
PABH cleft side	0.952693	0.000000*
LABH noncleft side	0.881951	0.000000*
PABH noncleft side	0.751228	0.000087*

*R* Spearman’s rank correlation coefficient, *LABH* labial alveolar bone height, *PABH* palatal alveolar bone height

*Statistically significant at *p* < 0.05

**Fig. 6:**
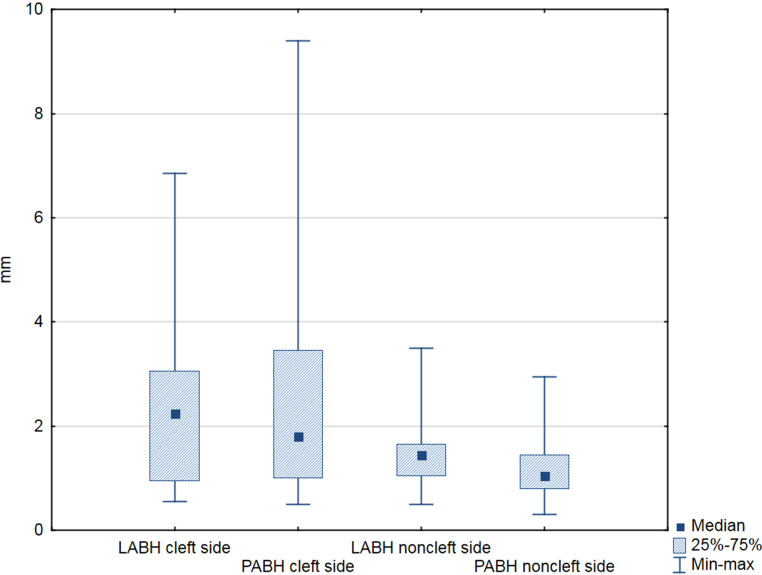
Box plot. *LABH* labial alveolar bone height, *PABH* palatal alveolar bone height, *mm* millimeters, *25–75%* 25th–75th percentile, *Min–max* Minimum–maximum Boxplot. *LABH* labiale Alveolarknochenhöhe, *PABH* palatinale Alveolarknochenhöhe, *mm *Millimeter, *25–75%* 25.–75. Perzentile, *Min–max* Minimum–Maximum

The Wilcoxon signed-rank test showed statistically significant (*p* < 0.05) differences between the cleft and noncleft side measurements. The cleft side LABH and PABH distances were significantly greater than those for the controls. The mean LABH measurement in the cleft region was 2.3 ± 1.47 mm, compared with 1.55 ± 0.8 mm for the noncleft region. The mean PABH measurements were 2.64 ± 2.57 and 1.23 ± 0.69 mm, respectively. The alveolar bone height measurements showed high interindividual variability on the experimental side (Fig. 6; Table 4).

**Table 4:** Intergroup comparisons Vergleiche zwischen den Gruppen

	Cleft side			Noncleft side			Mean difference (SD)	Median difference	*p*
	Mean (SD)	Median	Min–max	Mean (SD)	Median	Min–max			
LABH, mm	2.3 (1.47)	2.25	0.55–6.85	1.55 (0.8)	1.45	0.5–3.5	0.75 (1.33)	0.2	0.00636*
PABH, mm	2.64 (2.57)	1.8	0.5–9.4	1.23 (0.69)	1.05	0.3–3.0	1.41 (2.08)	0.65	0.00176*

SD standard deviation, *mm* millimeters, *Min–max* Minimum–maximum, *LABH* labial alveolar bone height, *PABH* palatal alveolar bone height

*Statistically significant at *p* < 0.05

A bootstrapping analysis (1 million repetitions) demonstrated that for the commonly applied significance level of 0.05 the power of the test with H0 (both datasets come from the distribution of the control measurements) vs. H1 (the difference between the measurements is as observed in the data) was higher than 95% on the labial and higher than 99% on the palatal side of the maxillary central incisors’ roots. Thus, reliability of the obtained results was proven.

## Discussion

The CBCT examination with a FOV of 5 cm × 5 cm provides detailed information about the cleft side and the corresponding unaffected part of the maxilla. The presented method of alveolar crest height measurements is useful and reproducible. The null hypothesis was rejected. The maxillary central incisors in UCLP patients did not have the same alveolar bone heights on the cleft and noncleft sides. There was a tendency to the presence of dehiscences on the cleft side.

Decreasing the CBCT voxel size from 0.4 to 0.25 mm can improve the accuracy of alveolar bone linear measurements. It provides clearer images, easier identification of the alveolar crests, and results closer to the gold standard (direct measurements) [24]. Therefore, the voxel size adopted in this study was adequate. However, examination with 0.2 mm voxel size provides on average spatial resolution of 0.4 mm. As a result, objects with a minimum 0.4 mm distance can be distinguished [2]. The spatial resolution is also affected by a scatter lever increasing with FOV size. The recommended reduction of FOV [17] was used in this study. It was smaller than in other papers assessing alveolar bone morphology in CLP patients [12, 15, 18, 25–28]. Reduction of the FOV size also results in an expected reduction of the radiation dose. This approach is in line with the ALARA (as low as reasonably achievable) principle. ALARA involves maintaining exposures to radiation as far below the dose limits as practical, while being consistent with the purpose for which the activity is undertaken [32]. Due to the smaller FOV size in this study than in the paper of Yatabe et al. [27] amounting to 16 cm × 6 cm, comparative bone thickness measurements with the described reorientation according to the molar trifurcation were not possible.

In cases of healthy periodontal structure, alveolar bone crest is positioned about 1 mm below the cementoenamel junction. If there is a lack of bone coverage on the cervical surface of tooth roots, dehiscences are present [6]. A drawback to CBCT is a documented underestimation of the bone volume [14, 24]. As a result, the critical value for the detection of a dehiscence on the CBCT was defined in the literature to be 2 mm [23]. To avoid underestimation of this size, no threshold of the bone thickness to identify and set the alveolar bone crest point was adopted in this study.

Previous studies also proved that CBCT examination is an adequate and reproducible method for the alveolar bone height assessment in CLP patients [8, 27, 28]. Yatabe et al. [27] did not obtain statistically significant differences (*p* > 0.05) in the palatal alveolar bone height measurements between cleft and noncleft sides in the incisors’ region. Furthermore, these authors did not obtain high interindividual variability in their cleft side measurements group. Opposite results were obtained on the labial surface. Study group selection might have had impacted these results. The authors examined 8 UCLA and 22 complete UCLP patients. The presence of the former group might have influenced the differences between both studies. Patients with UCLA tend to have a partial congenital bone continuity on the palatal side of the alveolus [29]. As opposed to their UCLP group, the authors did not differentiate whether the UCLA group consisted of incomplete and/or complete UCLA patients. However, this fact does not explain lower interindividual variability in this group. The variability difference might be due to more careful insertion of the bone grafts towards the palatal direction of the cleft. On the other hand, the above-mentioned study group consisted solely of patients treated with canine mesialization into the cleft area, resulting in contact between the canine and central incisor. This procedure is not possible in the absence of a bone bridge, with disrupted cleft fragments, or when the bone bridge is generally of poor quality. Therefore, the presented results refer to a particular group of patients and for this reason cannot be used for a general assessment [21]. Ercan et al. [8] utilized CBCT images performed before alveolar bone grafting for labial alveolar bone height measurements. Their results (cleft side LABH: 2.34 ± 1.09 mm, noncleft side LABH: 1.53 ± 0.69 mm) were similar to those obtained in this study. On the other hand, our results counter those described by Buyuk et al. [5]. They found that the prevalence of labial dehiscences on the noncleft side was almost as high as that on the cleft side. These authors evaluated only the presence or lack of a labial dehiscence without the comparison of linear measurements.

In this study, the cleft-adjacent maxillary central incisors presented more apically displaced alveolar bone crests on the labial and palatal sides of the roots than the controls. It seems that the clinical significance of this results should be evaluated with regard to value of the mean biological width (the dimension of the soft tissue, which is attached to the portion of the tooth coronal to the crest of the alveolar bone), which amount to 2.04 mm [11]. Considering this order of magnitude, mean differences (especially on the palatal side) are clinically significant. High interindividual variability of the obtained results indicates a need for routine alveolar bone height measurements in CLP patients before further treatment planning. The occurrence of prominent alveolar bone height differences should be taken under careful consideration during clinical procedures. Dehiscences are one of the factors, which predispose to gingival recession [6]. Differences in the gingival height above 1.5 to 2 mm are easily recognized both by dentists and by laypeople [13]. To obtain optimal esthetic results, especially in patients with gingival exposure when smiling, the cleft side central incisor may require orthodontic extrusion and crown length correction after vertical tooth movement. In cases requiring prosthetic restorations, pink tissue porcelain may be used. Inferior bony support and potential gingival recessions may increase the demand for both surgical and nonsurgical periodontal intervention on the cleft side. Moreover, patients with complete UCLP treated with fixed orthodontic appliances demonstrated a higher incidence of external apical root resorption on the cleft side maxillary anterior teeth than on the noncleft side [3]. Both root resorption and apically positioned bone crests on the cleft side decrease the bony support of the cleft adjacent maxillary central incisors. The smaller periodontal ligament surface will be loaded by greater pressure initiated by a defined amount of orthodontic force. Apically positioned bone crest also means that the center of resistance of the tooth translocates in the same direction. In these cases, movement of cleft adjacent teeth requires lower forces and relatively higher moments of force to control the root position in the sagittal dimension.

A previous CBCT study with UCLP patients showed that SABG did not provide good bone morphology in most cases [22]. These results indicate a need for further reconstructive surgical procedures to enhance the bone bridge quality. The effect of SABG on the alveolar bone crest height on the labial and palatal sides of the central incisors is unclear. Reported results of this study were obtained from one-time point analysis without longitudinal comparison with measurements performed before SABG. Therefore, since the main aim of the cleft grafting is the bone bridge obtainment, not enough (or even no) bone could have been placed on the labial and palatal surfaces of the incisors. SABG might have not improved the level of the bone in the measurement sites or even exacerbated it due to the elevated osteoclastic activity after flap elevation [10].

Due to significant morphologic differences, no blinding was used for cleft and noncleft side measurements. One limitation of this study was that the rater was an orthodontist. Radiologists, periodontists, oral surgeons, and maxillofacial surgeons also perform alveolar bone height measurements. However, orthodontists routinely evaluate the cleft area for further orthodontic treatment planning.

The size of the study group was limited but acceptable, and it is comparable with the majority of study that assessed alveolar bone morphology with 3D x‑ray diagnostics on UCLP patients only [21]. Patients were qualified according to the eligibility criteria, irrespective of the graft results (presence and quality of the bone bridge) to obtain a general and nonselective assessment of the alveolar bone height. Moreover, the power calculation demonstrated the reliability of the measured results.

The follow-up interval was heterogeneous and potentially could have had an influence on the results because of the time required for bone remodeling and bone resorption after SABG. However, Feichtinger et al. [9] published the longest prospective observation time among all those that described 3D x‑ray diagnostics for assessing SABG treatment outcomes [21]. The study’s results showed that follow-up differences did not seem to exert a significant influence.

There is a need for further prospective studies to assess alveolar bone heights before and after SABG and to compare results obtained with CBCT with parameters from periodontal clinical examination, especially with clinical attachment loss (CAL) measurements. It also seems justified to examine the effects of guided tissue regeneration in CLP patients with bone defects after SABG.

## Conclusions

Cone-beam computed tomography provided detailed information about alveolar bone morphology in cleft lip and palate patients. The presented method of alveolar crest height measurements was useful and reproducible. The cleft-adjacent maxillary central incisors had more apically displaced alveolar bone crests on the labial and palatal sides of the roots than the controls. A higher prevalence of dehiscences was found on the cleft side. Reduced bone support should be taken under careful consideration during further treatment planning.

## Supplementary Information

Supplementary Table
