# Supplementary material for: Alveolar bone heights of maxillary central incisors in unilateral cleft lip and palate patients using cone-beam computed tomography evaluation
Source: J Orofac Orthop. 2021 Feb 5;82(3):198–208. doi: 10.1007/s00056-020-00276-w (PMC8076140; doi:10.1007/s00056-020-00276-w)
Supplement: Supplementary file 1 — Supplementary Table [file 56_2020_276_MOESM1_ESM.docx]

| Patient’s serial number | Cleft side | | Noncleft side | |
| --- | --- | --- | --- | --- |
|  | LABH [mm] | PABH [mm] | LABH [mm] | PABH [mm] |
| 1 | 2.65 | 1.05 | 1.65 | 1.3 |
| 2 | 0.55 | 2 | 1.05 | 0.8 |
| 3 | 0.95 | 1.15 | 1 | 0.8 |
| 4 | 1.95 | 1.1 | 1.2 | 0.45 |
| 5 | 0.65 | 0.5 | 0.5 | 0.7 |
| 6 | 1.5 | 0.95 | 1.8 | 1.3 |
| 7 | 3.2 | 8.9 | 3.45 | 3 |
| 8 | 0.85 | 1 | 0.55 | 0.3 |
| 9 | 0.9 | 0.85 | 0.85 | 0.9 |
| 10 | 2.85 | 1.95 | 1.5 | 1.05 |
| 11 | 1 | 2.75 | 1.1 | 1.25 |
| 12 | 2.25 | 3.45 | 2.15 | 2.6 |
| 13 | 3.05 | 0.6 | 1.65 | 1.05 |
| 14 | 1.75 | 4 | 1.55 | 1.05 |
| 15 | 1.5 | 0.85 | 1 | 0.75 |
| 16 | 3.7 | 9.4 | 3.5 | 2.45 |
| 17 | 2.4 | 5.1 | 1.55 | 1 |
| 18 | 2.7 | 5.05 | 2.55 | 1.55 |
| 19 | 3.65 | 0.95 | 1.45 | 0.75 |
| 20 | 3.5 | 2 | 1.15 | 1.45 |
| 21 | 6.85 | 1.8 | 1.35 | 1.35 |

Supplementary Table. Arithmetic means of both measurement series in all patients.

Measurements were performed twice with an interval of at least 2 months. Arithmetic means were used for further comparative analyses.

*LABH* – labial alveolar bone height, *PABH* – palatal alveolar bone height, *mm* – millimeters
